# Triple‐Phase Computed Tomography for Differentiating Canine Cutaneous and Subcutaneous Mast Cell Tumors From Benign Lesions: A Size‐Based Evaluation

**DOI:** 10.1111/vru.70256

**Published:** 2026-09-17

**Authors:** Motomu Morishita, Takuya Kusaka, Mayumi Murata, Toshiyuki Tanaka

**Affiliations:** ^1^ Veterinary Medical Center School of Veterinary Science Osaka Metropolitan University Osaka Japan; ^2^ Laboratory of Veterinary Advanced Diagnosis and Treatment School of Veterinary Science Osaka Metropolitan University Osaka Japan

**Keywords:** delayed enhancement, diagnostic imaging, dynamic CT, multiphase, tumor characterization

## Abstract

Canine cutaneous and subcutaneous mast cell tumors (MCTs) have highly variable clinical presentation, often mimicking benign lesions. This retrospective case–control study aimed to identify triple‐phase computed tomography (CT) features that distinguish MCTs from benign lesions, with an emphasis on tumor size. Twenty‐three MCTs (10 large [≥3 cm]; 13 small [<3 cm]) and seven benign lesions were included. Qualitative features and CT attenuation values were compared among three groups by size and type. Large MCTs were primarily characterized by qualitative CT features. Significant differences among the three groups were observed in heterogeneous enhancement patterns, feeding vessels, infiltration, necrosis, and fat stranding. These features were most frequently observed in large MCTs. In contrast, small MCTs lacked these features but showed significantly higher CT attenuation values in the portal venous and equilibrium phases than benign lesions (*p* = 0.025 and 0.034, respectively). Receiver operating characteristic curve analysis for discriminating small MCTs from benign lesions yielded optimal cutoff values of 68.7 Hounsfield units (HU) for the portal venous phase (area under the curve [AUC] = 0.88; sensitivity, 92%; specificity, 71%) and 74.7 HU for the equilibrium phase (AUC = 0.85; sensitivity, 92%; specificity, 57%). In conclusion, despite the limited sample size, these findings suggest that triple‐phase CT has clinical utility for differentiating MCTs from benign lesions. Qualitative patterns are particularly informative for large MCTs, whereas CT attenuation values in the portal venous and equilibrium phases may serve as objective adjunct indicators for identifying small MCTs that lack distinct morphological features.

**Abbreviations**
CT: computed tomography
HU: Hounsfield units
MCTs: mast cell tumors

## 1 Introduction

Mast cell tumors (MCTs) are among the most common cutaneous neoplasms in dogs, accounting for 16%–21% of all skin tumors [1]. The clinical presentation of cutaneous and subcutaneous MCTs is highly variable and often mimics that of benign lesions on physical examination [1]. Although fine‐needle aspiration (FNA) is primarily used for diagnosis, histopathological grading is essential for predicting biological behaviors [2, 3, 4]. According to World Health Organization guidelines, clinical staging is determined by evaluating lymph node involvement, multiplicity, tumor size, degree of infiltration, and distant metastasis [5]. Notably, MCTs exceeding 3 cm in diameter are associated with more aggressive biological behavior [6].

Although computed tomography (CT) has certain limitations in accurately delineating tumor margins and detecting metastases, it is increasingly used as an adjunct tool for diagnostic evaluation and staging [1, 7, 8, 9]. CT may also be used to identify clinically occult lesions. One study detected incidental MCTs in 32% of dogs that underwent CT for known MCTs [10]. Limited information is available regarding the CT characteristics of canine cutaneous and subcutaneous MCTs, and there are currently no established imaging criteria for distinguishing them from other benign lesions that share similar clinical presentations [9, 10].

Triple‐phase CT (arterial, portal venous, and equilibrium phases) allows the assessment of temporal changes in enhancement patterns, which may improve the characterization of lesions with distinct hemodynamic and internal architectures [11, 12]. Although its utility in differentiating malignant from benign lesions has been demonstrated in the canine liver and spleen [13, 14], published CT findings for MCTs have primarily been limited to single‐phase evaluations in the portal venous or equilibrium phases [9, 10]. Triple‐phase CT findings may reflect complex histological features such as angiogenesis and fibrosis, thereby facilitating early detection and guiding treatment planning.

This study aimed to identify triple‐phase CT features that differentiate canine MCTs from benign lesions, with particular emphasis on tumor size, in large (≥3 cm) and small MCTs (<3 cm). We hypothesized that triple‐phase CT would reveal size‐dependent imaging characteristics that could differentiate MCTs from benign lesions.

## 2 Materials and Methods

This retrospective case–control study used clinical data obtained with informed consent from owners for diagnostic procedures, treatment, and research purposes. Because all evaluations were part of routine clinical care, the requirement for formal ethical approval was waived by the institutional review committee. All procedures complied with institutional guidelines for animal care and use.

### 2.1 Case Selection

The medical records of dogs diagnosed with histopathologically confirmed MCTs at our institution between October 2018 and May 2025 were reviewed. The inclusion criteria for the MCT group were (1) the presence of cutaneous or subcutaneous MCTs and (2) inclusion of the lesion within the triple‐phase CT field of view. In the control group, dogs with benign lesions were included if they met the following criteria: (1) the presence of cutaneous or subcutaneous nodules or masses, (2) definitive histopathological diagnosis of a benign lesion, and (3) inclusion of the lesion within the triple‐phase CT field of view. Lipomas were excluded from the study because they were readily distinguishable from other soft‐tissue lesions based on their characteristic fat‐equivalent CT attenuation [15]. Case selection was performed by a veterinarian with 7 years of clinical experience (M.M.).

### 2.2 Medical Record Review

Clinical data, including breed, sex, age, body weight, tumor location, and histopathology reports, were extracted from medical records.

### 2.3 CT Protocol

CT scans were performed using either a 16‐slice CT scanner (Activion 16; Canon Medical Systems, Otawara, Japan) or an 80‐slice CT scanner (Aquilion Lightning; Canon Medical Systems) in helical scan mode. All dogs were placed under general anesthesia in either the supine or prone position, depending on the clinical circumstances. CT was performed without external compression to minimize deformation of the lesions. The scan parameters for the Activion 16 included a pitch of 0.9, rotation time of 0.75 s, 120 kV, and 100–150 mA. For the Aquilion Lightning, the parameters were a pitch of 0.8, rotation time of 0.6 s, 120 kV, and 100–150 mA. Raw data were reconstructed with a slice thickness of 0.5 and 2.0 mm using a soft‐tissue reconstruction algorithm. For contrast‐enhanced imaging, a nonionic contrast medium (300 mgI/mL; Ioverin 300; Teva Pharma Japan, Aichi, Japan) was administered at a dose of 600 mgI/kg (2 mL/kg) via a cephalic vein catheter using a power injector with a fixed injection duration of 20 s. Triple‐phase scans were acquired at the following time points: arterial phase (20 s after contrast injection), portal venous phase (60 s), and equilibrium phase (180 s).

### 2.4 Image Analysis

Image analysis was performed on a DICOM workstation (OsiriX v11.0.3; Pixmeo, Geneva, Switzerland) using soft tissue (WL 40 Hounsfield units [HU] and WW 350 HU) and bone (WL 300 HU and WW 1500 HU) window settings. The bone window was used to identify internal mineralization. Two experienced veterinary radiologists (M.M. and T.K., with 7 and 6 years of experience, respectively), blinded to the histopathological diagnoses, independently reviewed the images in a randomized order. Discrepancies in qualitative assessments were resolved by a third veterinary radiologist (T.T., with 21 years of experience) acting as an arbitrator [16]. All measurements were performed using a standardized protocol.

The following qualitative CT features were recorded: shape (round, oval, flat, or irregular); periphery (smooth or irregular); margins (well‐defined or ill‐defined); feeding vessels (present or absent); necrosis (present or absent); mineralization (present or absent); fat stranding (present or absent); infiltration (present or absent); and contrast enhancement pattern for each phase (heterogeneous, homogeneous, or no contrast enhancement). The shape was subjectively categorized on the basis of a visual assessment of the overall contour and symmetry on multiplanar images. The periphery was classified as smooth or irregular, based on the uniformity of the outer border. Margins were categorized as well‐defined or ill‐defined according to the clarity of demarcation from adjacent tissues. Feeding vessels were defined as distinct branches originating from or directly leading to the lesions on contrast‐enhanced imaging. Necrosis was defined as a focal area of low attenuation within the lesion showing no contrast enhancement across all post‐contrast planes. Mineralization was defined as the presence of high‐attenuation foci within the lesion on CT images, particularly in the bone‐window settings. Infiltration was defined as extension of the lesion into adjacent tissues with a loss of normal tissue planes. Fat stranding was defined as increased attenuation and linear or reticular soft tissue changes in the surrounding fat. Lesions were classified as homogeneous if the enhancement was visually uniform and heterogeneous if they contained areas of differing attenuation, including low‐attenuation areas or necrosis. No contrast enhancement was defined as an increase of less than 20 HU on post‐contrast images compared to pre‐contrast images [17]. Findings that could not be determined were recorded as not applicable.

The following quantitative CT features were recorded: lesion size (maximum diameter) and CT attenuation values (HU) in the pre‐ and post‐contrast phases. Lesion size was defined as the maximum diameter measured on preoperative CT images using multiplanar reconstructed images in any of three orthogonal planes. Lesion classification was based solely on these CT measurements. To measure attenuation on the CT images, freehand regions of interest (ROIs) were placed along the inner margin of the lesion on the same transverse image matched across all phases. ROIs were manually placed to maximize lesion coverage, while carefully excluding visible vessels and necrotic regions to minimize measurement bias. The ROI measurements for each image were repeated three times, and the mean attenuation values were calculated.

### 2.5 Statistical Analysis

Statistical analyses were performed using EZR v1.61 [18]. Normality was assessed using the Shapiro–Wilk test. As all continuous variables followed a normal distribution, they are reported as mean ± standard deviation; categorical variables are expressed as frequencies and percentages. Data, recorded as “not applicable,” were excluded from analysis. Analyses compared three groups: large MCTs (≥3 cm), small MCTs (<3 cm), and benign lesions, using a size cutoff of 3 cm based on previous reports [6].

Overall comparisons among the three groups were performed using Fisher's exact test for qualitative features and one‐way analysis of variance for quantitative CT attenuation values. For qualitative variables, only the overall *p* values from Fisher's exact test are reported, and no post hoc pairwise comparisons were performed. When overall significance was identified in CT attenuation values, pairwise comparisons were performed using Bonferroni correction. For specific qualitative features, the categories were dichotomized (shape: round/oval vs. other shapes; enhancement pattern: heterogeneous vs. non‐heterogeneous).

ROC curve analysis was performed to determine the optimal cutoff values for quantitative parameters that differed significantly between small MCTs and benign lesions. Cutoff values were selected to maximize specificity while maintaining a sensitivity of ≥90% to prioritize clinical detection of MCTs and minimize false negatives. The area under the curve (AUC), sensitivity, and specificity are reported with 95% confidence intervals (CIs). Statistical significance was defined as *p* < 0.05.

## 3 Results

### 3.1 Study Population and Signalment

The signalment data for dogs with MCTs and benign lesions are summarized in Table 1. A total of 22 dogs with 23 MCTs were enrolled; 1 dog presented with two distinct tumors. Among the 23 MCTs, 10 were categorized as large MCTs (≥3 cm) and 13 as small MCTs (<3 cm) on the basis of their maximum diameter. The histopathological grading of the nine cutaneous MCTs, according to the Patnaik and Kiupel systems [3, 4], was as follows: Three were Grade II/low‐grade, one was Grade II/high‐grade, and five were Grade III/high‐grade. Indications for CT included staging and treatment planning (13 dogs), surgical planning (4 dogs), evaluation of postoperative recurrence (3 dogs), radiation therapy planning (1 dog), and evaluation of an intra‐abdominal mass (1 dog). Clinical signs at the time of presentation included ataxia (2 dogs), lethargy (1 dog), and pruritus (1 dog), whereas the remaining 18 dogs were asymptomatic. Clinicopathological abnormalities included leukocytosis and elevated C‐reactive protein in four dogs. FNA was performed within 3 weeks of CT in 10 dogs. The interval between FNA and CT ranged from 7 to 20 days (12 median days). At the time of CT, seven dogs were receiving oral prednisolone (0.2–1.0 mg/kg, every 24 h) and two were receiving oral toceranib (2.0–3.0 mg/kg, every 48 h).

**TABLE 1:** Signalment data for dogs with MCTs and benign lesions.

		MCTs *n* = 23 (22 dogs)	Benign lesions *n* = 7 (seven dogs)
Breed (dogs)		Labrador Retriever (5) Toy Poodle (5) Chihuahua (2) French Bulldog (2) Pug (2) Schnauzer (2) Others (4)^a^	Bernese Mountain Dog (1) French Bulldog (1) Golden Retriever (1) Labrador Retriever (1) Shih Tzu (1) Toy Poodle (1) Welsh Corgi Pembroke (1)
Sex (dogs)	Neutered male Intact male Spayed female Intact female	3 (14%) 5 (23%) 11 (50%) 3 (14%)	3 (43%) 1 (14%) 0 (0%) 3 (43%)
Mean age ± SD (years)		11.1 ± 2.7 (range 7.1–15.3)	9.9 ± 2.7 (range 7.7–14.9)
Mean weight ± SD (kg)		12.6 ± 9.9 (range 2.8–36.0)	19.4 ± 12.8 (range 3.8–36.2)
Histopathological site (*n*)	Cutaneous	9 (39%)	3 (43%)
	Subcutaneous	14 (61%)	4 (57%)
Anatomical location (*n*)	Head	5 (22%)	0 (0%)
	Neck	3 (13%)	0 (0%)
	Trunk	2 (9%)	3 (43%)
	Forelimb excluding digits	4 (17%)	1 (14%)
	Hindlimb excluding digits	2 (9%)	3 (43%)
	Digits	4 (17%)	0 (0%)
	Inguinal/Perineal	2 (9%)	0 (0%)
	Tail	1 (4%)	0 (0%)

Abbreviations: MCT, mast cell tumor; SD, standard deviation.

^a^Others include four distinct breeds (one each).

The control group consisted of seven dogs, each with a single benign lesion. These lesions included apocrine cysts, cavernous hemangiomas, fibroepithelial polyps, fibromas, fibrous hamartomas, sebaceous adenomas, and sebaceous hyperplasia. All benign lesions were surgically excised and histopathologically confirmed.

### 3.2 Qualitative CT Findings

**FIGURE 1:**
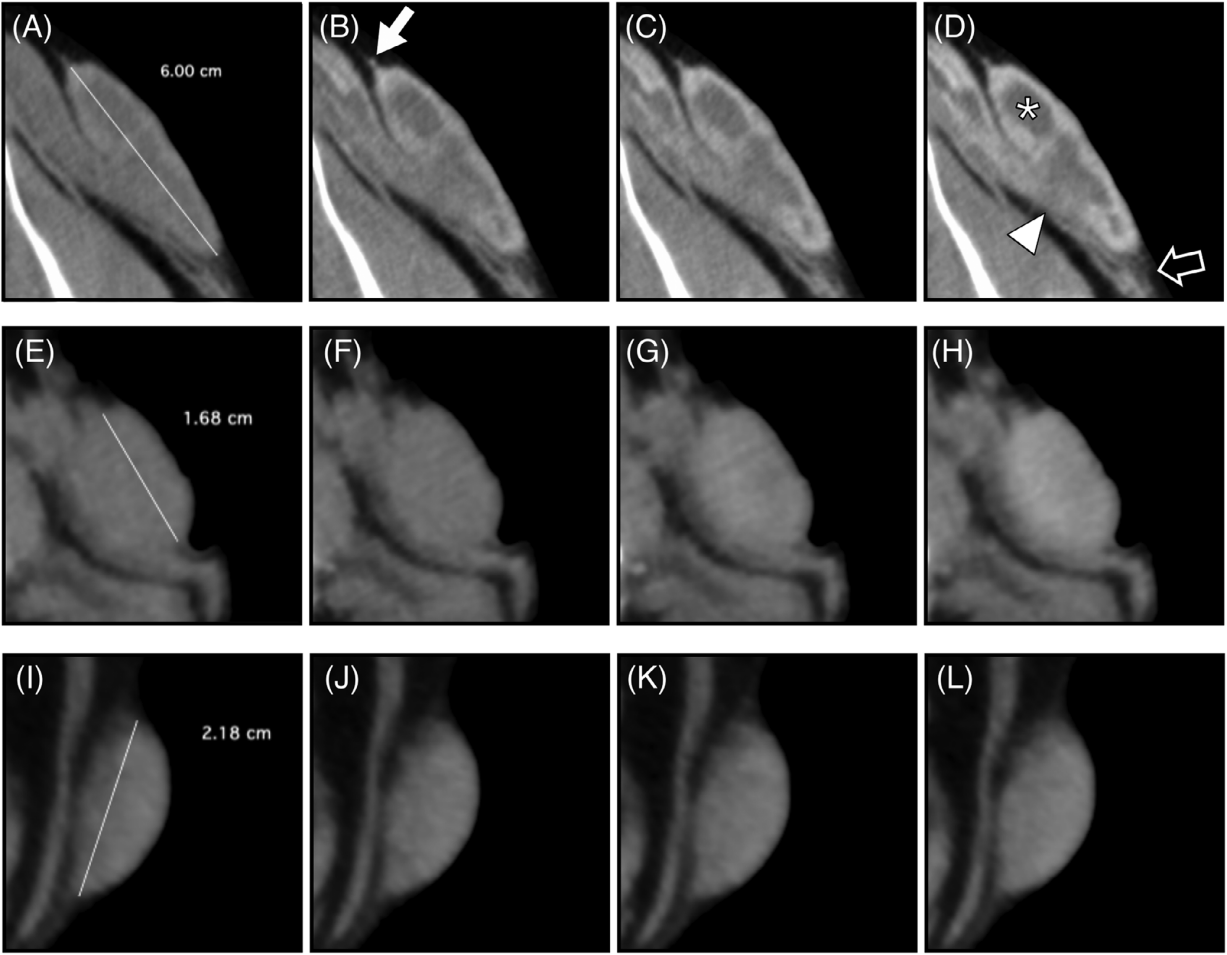
Triple‐phase CT of a large MCT (≥3 cm, A–D), small MCT (<3 cm, E–H), and a benign cavernous hemangioma (I–L) in three dogs. Images represent pre‐contrast (left column), arterial phase (second column), portal venous phase (third column), and equilibrium phase (right column). Maximum lesion diameters (cm) are indicated on pre‐contrast images. In the large MCT (A–D), marked heterogeneous enhancement, a prominent feeding vessel (B, arrow), extensive necrosis (D, asterisk), infiltration (D, arrowhead), and fat stranding (D, open arrow) are visible across the contrast‐enhanced phases. Note the relatively higher CT attenuation in the small MCT (G and H) than in the benign lesion (K and L) during the portal venous and equilibrium phases. All images are displayed using a soft tissue window (WL = 40 HU, WW = 350 HU). CT, computed tomography; HU, Hounsfield units; MCT, mast cell tumor.

The qualitative CT findings of large MCTs (*n* = 10), small MCTs (*n* = 13), and benign lesions (*n* = 7) (Figure 1) are summarized in Table 2. The presence of feeding vessels differed significantly between the groups (*p* = 0.019). Large MCTs showed the highest prevalence of feeding vessel presence (100%), followed by small MCTs (77%) and benign lesions (43%), suggesting that feeding vessels tend to be more frequently observed in larger tumors. There was a significant overall difference in necrosis among the three groups (*p* = 0.006). Necrosis was most frequently observed in large MCTs (60%), whereas it was uncommon in small MCTs (8%) and was not detected in benign lesions (0%). Infiltration showed a significant overall difference among the three groups (*p* < 0.001). Infiltration was observed only in large MCTs (60%) and was absent in both small MCTs and benign lesions. Fat stranding also differed significantly among the three groups (*p* = 0.020). It was most frequently identified in large MCTs (60%), less commonly identified in small MCTs (15%), and absent in benign lesions (0%).

**TABLE 2:** Qualitative CT findings of MCTs and benign lesions.

		*MCTs*	*MCTs*	*Benign lesions*	*p* value (overall)
		Large (≥3 cm) (*n* = 10)	Small (<3 cm) (*n* = 13)	(*n* = 7)	*p* value (overall)
Shape	Round	3 (30%)	1 (8%)	2 (29%)	0.326
	Oval	2 (20%)	3 (23%)	3 (43%)	
	Flat	1 (10%)	3 (23%)	0 (0%)	
	Irregular	4 (40%)	5 (39%)	2 (29%)	
	N/A	0 (0%)	1 (8%)	0 (0%)	
Periphery	Smooth	8 (80%)	11 (85%)	7 (100%)	0.651
	Irregular	2 (20%)	2 (15%)	0 (0%)	
Margins	Well‐defined	5 (50%)	4 (31%)	6 (86%)	0.087
	Ill‐defined	5 (50%)	9 (69%)	1 (14%)	
Feeding vessels	Present	10 (100%)	10 (77%)	3 (43%)	0.019
	Absent	0 (0%)	3 (23%)	4 (57%)	
Necrosis	Present	6 (60%)	1 (8%)	0 (0%)	0.006
	Absent	4 (40%)	12 (92%)	7 (100%)	
Mineralization	Present	0 (0%)	0 (0%)	0 (0%)	>0.999
	Absent	10 (100%)	13 (100%)	7 (100%)	
Fat stranding	Present	6 (60%)	2 (15%)	0 (0%)	0.020
	Absent	4 (40%)	9 (69%)	7 (100%)	
	N/A	0 (0%)	2 (15%)	0 (0%)	
Infiltration	Present	6 (60%)	0 (0%)	0 (0%)	**<0.001**
	Absent	4 (40%)	11 (85%)	7 (100%)	
	N/A	0 (0%)	2 (15%)	0 (0%)	
Contrast enhancement pattern	Contrast enhancement pattern				
Arterial	Heterogeneous	10 (100%)	4 (31%)	0 (0%)	**<0.001**
	Homogeneous	0 (0%)	5 (39%)	2 (29%)	
	NCE	0 (0%)	4 (31%)	5 (71%)	
Portal venous	Heterogeneous	10 (100%)	7 (54%)	0 (0%)	**<0.001**
	Homogeneous	0 (0%)	6 (46%)	3 (43%)	
	NCE	0 (0%)	0 (0%)	4 (57%)	
Equilibrium	Heterogeneous	10 (100%)	7 (54%)	0 (0%)	**<0.001**
	Homogeneous	0 (0%)	6 (46%)	3 (43%)	
	NCE	0 (0%)	0 (0%)	4 (57%)	

*Note: p* values for overall comparisons among the three groups were calculated using Fisher's exact test. For statistical analysis, the shapes were compared as round/oval vs. other shapes, and the enhancement patterns were heterogeneous vs. non‐heterogeneous. Bold *p* values indicate statistical significance (*p* < 0.05).

Abbreviations: MCT, mast cell tumor; N/A, not applicable; NCE, no contrast enhancement; SD, standard deviation.

Contrast enhancement patterns across all phases showed highly significant differences among the three groups (all *p* < 0.001). All large MCTs (100%) exhibited heterogeneous enhancement patterns across all phases. In the arterial phase, this heterogeneous pattern was more frequent in large MCTs than in small MCTs (31%) and was absent in benign lesions (0%). In the portal venous and equilibrium phases, heterogeneous enhancement was observed in 54% of the small MCTs and was absent in benign lesions.

### 3.3 Quantitative CT Findings

**FIGURE 2:**
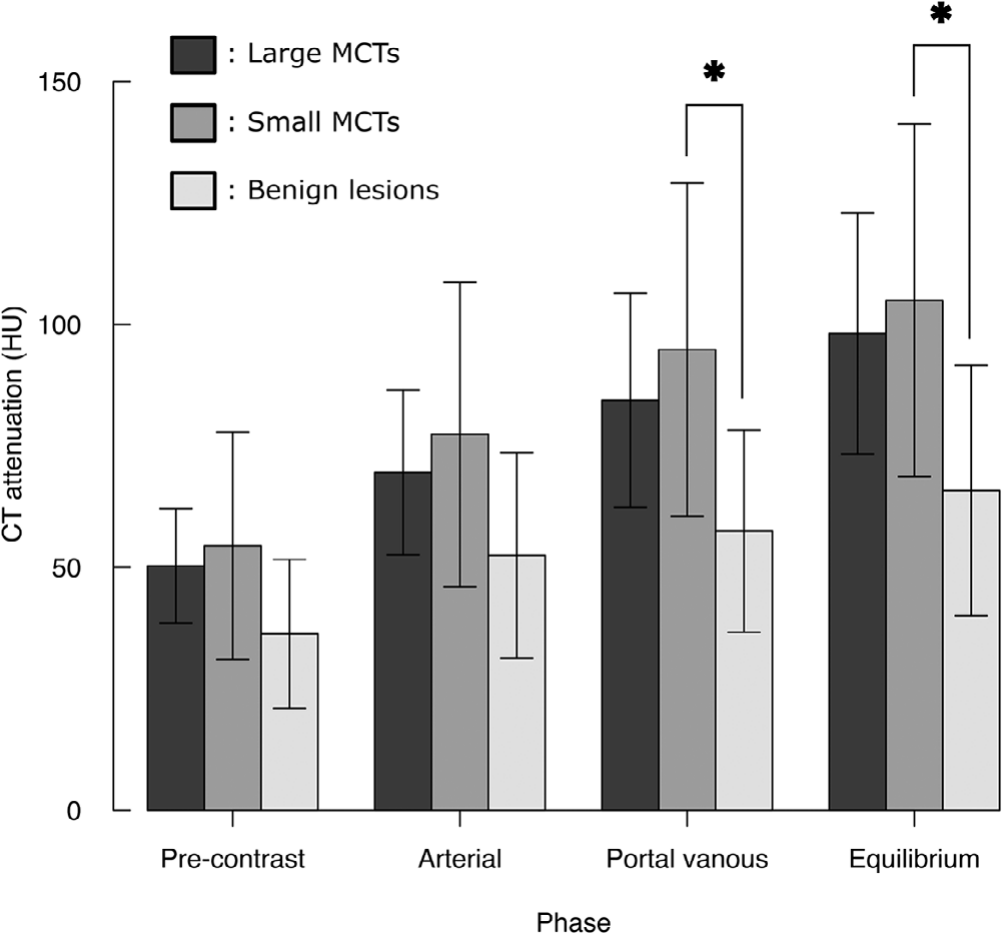
Comparison of CT attenuation values among large MCTs (≥3 cm), small MCTs (<3 cm), and benign lesions across all phases. Significant differences were observed between small MCTs and benign lesions in the portal venous and equilibrium phases. Error bars represent the standard deviation. Asterisks indicate significant differences (*p* < 0.05) on the basis of pairwise comparisons using Bonferroni correction. CT, computed tomography; HU, Hounsfield units; MCT, mast cell tumor; SD, standard deviation.

The maximum diameters of large MCTs, small MCTs, and benign lesions were 5.1 ± 1.3, 1.8 ± 0.6, and 1.8 ± 1.3 cm, respectively. Mean CT attenuation values for large MCTs, small MCTs, and benign lesions were 50.3 ± 11.8, 54.5 ± 23.4, and 36.3 ± 15.3 HU in the pre‐contrast phase; 69.5 ± 17.0, 77.4 ± 31.4, and 52.4 ± 25.9 HU in the arterial phase; 84.4 ± 22.1, 94.8 ± 34.3, and 57.4 ± 20.8 HU in the portal venous phase; and 98.1 ± 24.8, 105.0 ± 36.3, and 65.8 ± 25.8 HU in the equilibrium phase (Figure 2). Significant overall differences among the three groups were observed in the portal venous (*p* = 0.028) and equilibrium phases (*p* = 0.033). Pairwise comparisons revealed that small MCTs had significantly higher CT attenuation values than did benign lesions in the portal venous (*p* = 0.025) and equilibrium phases (*p* = 0.034).

ROC curve analysis was performed to evaluate diagnostic performance in discriminating small MCTs from benign lesions. In this study cohort, the optimal cutoff values were determined to be 68.7 HU for the portal venous phase (AUC = 0.88; 95% CI: 0.72–1.00; sensitivity, 92%; specificity, 71%) and 74.7 HU for the equilibrium phase (AUC = 0.85; 95% CI: 0.65–1.00; sensitivity, 92%; specificity, 57%).

## 4 Discussion

This study suggests that triple‐phase CT provides size‐dependent diagnostic indicators for differentiating canine MCTs (both cutaneous and subcutaneous) from benign lesions. MCTs were stratified by tumor size into large (≥3 cm) and small (<3 cm) groups to explore the potential size‐related differences in CT characteristics [6]. Large MCTs were primarily characterized by their qualitative CT features. Significant overall differences among the three groups were observed in the heterogeneous enhancement patterns across all phases, feeding vessels, infiltration, necrosis, and fat stranding. These features were most frequently observed in large MCTs. In contrast, small MCTs (<3 cm) tend to lack these characteristic qualitative features. However, quantitative analysis revealed that small MCTs had significantly higher CT attenuation values in the portal venous and equilibrium phases than benign lesions. These findings suggest that qualitative assessments are particularly informative for large MCTs, whereas quantitative thresholds may be more reliable for identifying small MCTs.

Heterogeneous enhancement is commonly associated with malignant tumors and reflects histopathological features, such as angiogenesis, variations in cellular density, and necrosis [19, 20]. In the present study, large MCTs exhibited more heterogeneous enhancement than small MCTs and benign lesions. MCTs, particularly those of higher grades, are known to exhibit hemorrhage, edema, necrosis, and fibrosis [21]. These histopathological characteristics may have contributed to the heterogeneous enhancement observed on CT scans in this cohort. In contrast, approximately half of the small MCTs showed homogeneous enhancement, leading to imaging appearances that overlapped with those of benign lesions. This overlap suggests that qualitative enhancement patterns alone may be insufficient to reliably distinguish between smaller tumors. Although a previous CT study reported primarily homogeneous enhancement in MCTs, this discrepancy may reflect differences in tumor biological behavior, as that study included a higher proportion of lesions diagnosed solely by cytology and, therefore, may have enrolled more tumors with relatively low malignant potential [10].

The feeding vessels observed on CT may reflect vascular remodeling in response to the metabolic demands of the tumor [11]. The expression of vascular endothelial growth factor in MCTs is known to increase microvessel density, and macroscopically visible vessels detected on CT may represent either compensatory enlargement of preexisting vessels or neoangiogenesis driven by increased tumor blood flow [22, 23]. In the present study, large MCTs exhibited the highest prevalence of feeding vessels (100%), followed by small MCTs (77%) and benign lesions (43%). These findings are consistent with those of a previous study suggesting that tumor size is related to the appearance of feeding vessels on CT [10]. However, feeding vessels can also be observed in benign lesions because an increased vascular supply may occur in growing masses or inflammatory processes [11]. Collectively, these results indicated that feeding vessels are a common feature of larger MCTs, although their presence alone may not reliably distinguish smaller tumors from benign lesions.

Large MCTs frequently exhibit aggressive features, such as infiltration, necrosis, and fat stranding, which are more prominent than those in small MCTs and benign lesions. These features are often associated with aggressive biological behavior, which increases in frequency when MCTs exceed 3 cm in diameter [6]. In contrast, small MCTs exhibit fewer distinct qualitative features, making it difficult to distinguish them from benign lesions based solely on qualitative imaging findings.

Quantitative CT analysis revealed that the CT attenuation values in the portal venous and equilibrium phases were significantly higher in small MCTs than in benign lesions, whereas no significant difference was observed between large MCTs and benign lesions. Cutoff values were established to prioritize MCT detection by maintaining a sensitivity of ≥90% and minimizing false‐negative results. Although the portal venous phase yielded a slightly higher AUC, the CT attenuation values for MCTs peaked during the equilibrium phase, potentially increasing the lesion‐to‐background contrast. Therefore, evaluating both phases together may optimize the diagnostic performance. Although the specificity was relatively limited (57%–71%), this likely reflects a trade‐off inherent in prioritizing high sensitivity. These attenuation thresholds may serve as objective adjunctive indicators for clinicians; however, cytology or histopathology remains essential for a definitive diagnosis. The lack of a significant difference in attenuation values between large MCTs and benign lesions may reflect the reduced blood flow associated with decreased functional vascular density and increased interstitial fibrosis, resulting in diminished contrast enhancement [19, 20].

Notably, the MCTs in the present study reached peak attenuation during the equilibrium phase. In humans, delayed enhancement patterns have been associated with fibrous stroma and expanded extracellular spaces, as observed in intrahepatic cholangiocarcinoma [24]. Mast cells release various profibrotic mediators from their granules, including histamine and growth factors, potentially inducing intratumoral fibrosis and edema [21, 22]. This may facilitate retention of the contrast medium within the lesion [11, 24]. This mechanism may partially explain the delayed enhancement patterns observed in this study. Delayed peak enhancement also suggests a limitation of single‐phase imaging, as maximal tumor conspicuity may only be observed in later phases.

Most previous reports describing the CT findings of canine cutaneous and subcutaneous lesions have primarily focused on malignant tumors such as soft tissue sarcomas and MCTs [9, 10, 25], whereas reports describing the CT characteristics of benign cutaneous and subcutaneous lesions remain limited. In human medicine, benign lesions generally demonstrate relatively homogeneous contrast enhancement on CT [26]. In the present study, benign lesions demonstrated relatively homogeneous contrast enhancement, which is consistent with human findings. However, because multiple benign lesion types were analyzed in a single group, the ability to evaluate individual CT characteristics was limited. A Japanese epidemiological study reported that benign lesions comprised 43.3% of canine skin tumors (excluding lipomas), including benign follicular tumors (e.g., trichoblastoma, trichoepithelioma, 9.3%), benign sebaceous tumors (8.5%), hamartomas (4.3%), and hemangiomas (3.7%) [27]. Our study lacked benign follicular tumors, which are one of the most prevalent benign canine skin tumors; thus, our benign lesion composition may not fully represent the canine population. The CT characteristics of benign canine follicular tumors have not yet been reported. In humans, these tumors appear as well‐defined lesions with calcifications and show homogeneous to peripheral contrast enhancement on CT [28]. Therefore, similar imaging characteristics may also be observed in dogs. Further studies are needed to evaluate the CT characteristics of individual benign lesions using triple‐phase CT.

As most previous reports relied on single‐phase CT [9, 10], certain vascular and stromal characteristics may have been underestimated when evaluated at a single time point. This study highlights the added value of triple‐phase CT for depicting these features. Our findings provide clinicians with practical triple‐phase CT indicators for pre‐surgical planning and staging. Utilizing CT attenuation values in the portal venous and equilibrium phases may facilitate the detection of small MCTs that may have been overlooked during physical examination. Given the relatively high prevalence of MCTs (11%–14%) and their potential to occur anywhere in the body, including the head, digits, and extremities [1, 21, 29], whole‐body CT evaluation may be beneficial. Although our results are promising, larger studies are warranted to compare the triple‐phase CT performance with optimized single‐phase protocols. Despite the small sample size, statistically significant differences in CT attenuation values and qualitative features, even after multiplicity adjustment, demonstrated the clinical utility of triple‐phase CT. These findings suggest that multiphasic contrast‐enhanced CT may improve the diagnostic assessment of canine cutaneous and subcutaneous MCTs.

This study had several limitations. First, prior FNA may have influenced local imaging findings in some cases. Although the interval between FNA and CT was at least 7 days in all cases and any clinically relevant influence was likely minimal, subtle effects on surrounding fat attenuation or lesion CT attenuation values cannot be completely excluded. Second, some MCT patients were receiving prednisolone or toceranib, which may have affected tumor vascularity and contrast enhancement patterns, potentially leading to an underestimation of the diagnostic performance of triple‐phase CT. Third, the comparisons were limited to MCTs and benign lesions. Therefore, the findings cannot be directly extrapolated to differentiate MCTs from other malignant tumors with similar hemodynamic characteristics. Fourth, cutaneous and subcutaneous MCTs were analyzed collectively because CT cannot reliably distinguish their tissue origins, and histopathological grading systems are inconsistent for subcutaneous tumors, limiting tissue‐specific interpretation and correlation with tumor grade. Fifth, the small sample size prevented detailed comparisons among specific benign lesion types. Similarly, grouping various benign lesions into a single entity may obscure type‐specific triple‐phase CT characteristics. Finally, the 3‐cm tumor size cutoff and referral‐based case selection may have introduced a selection bias. Prospective studies with larger cohorts are required to validate these findings.

In conclusion, these findings suggest that heterogeneous enhancement, feeding vessels, infiltration, necrosis, and fat stranding are useful indicators to distinguish large MCTs from benign lesions. For small MCTs that tend to lack distinct morphological features, CT attenuation values in the portal venous and equilibrium phases may provide objective diagnostic indicators. Although these findings are encouraging, the small sample size warrants further large‐scale studies to validate these diagnostic indicators and to determine their broader clinical utility.

## Author Contributions

Conception and design: Motomu Morishita. Acquisition of data: Motomu Morishita, Takuya Kusaka, and Toshiyuki Tanaka. Analysis and interpretation of data: Motomu Morishita, Takuya Kusaka, and Toshiyuki Tanaka. Drafting the article: Motomu Morishita. Revising article for intellectual content: Takuya Kusaka, Mayumi Murata, and Toshiyuki Tanaka. Final approval of the completed article: Motomu Morishita, Takuya Kusaka, Mayumi Murata, and Toshiyuki Tanaka. Agreement to be accountable for all aspects of the work in ensuring that questions related to the accuracy or integrity of any part of the work are appropriately investigated and resolved: Motomu Morishita, Takuya Kusaka, Mayumi Murata, and Toshiyuki Tanaka.

## Disclosure

A portion of this work was previously presented at the Annual Meeting of the Japanese Society of Veterinary Diagnostic Imaging (Osaka, Japan; December 2025). This study was reported in accordance with the STROBE guidelines.

## Conflicts of Interest

The authors declare no conflicts of interest.

## Data Availability

The data supporting the findings of this study are available from the corresponding author upon reasonable request.
